# A novel atlas of gene expression in human skeletal muscle reveals molecular changes associated with aging

**DOI:** 10.1186/s13395-015-0059-1

**Published:** 2015-10-09

**Authors:** Jing Su, Carl Ekman, Nikolay Oskolkov, Leo Lahti, Kristoffer Ström, Alvis Brazma, Leif Groop, Johan Rung, Ola Hansson

**Affiliations:** European Molecular Biology Laboratory—European Bioinformatics Institute, Wellcome Trust Genome Campus Hinxton, Cambridge, CB10 1SD UK; Lund University Diabetes Center, Department of Clinical Sciences, Diabetes and Endocrinology, Skåne University Hospital Malmö, Lund University, Malmö, 20502 Sweden; Department of Veterinary Biosciences, University of Helsinki, PO Box 66, FI-00014 Helsinki, Finland; Swedish Winter Sports Research Centre, Department of Health Sciences, Mid Sweden University, SE-83125 Östersund, Sweden; Department of Immunology, Genetics and Pathology, Science for Life Laboratory, Rudbeck Laboratory, Uppsala University, 751 85 Uppsala, Sweden

**Keywords:** Skeletal muscle, Expression, Microarray, Aging, Mitochondrial dysfunction, Exercise

## Abstract

**Background:**

Although high-throughput studies of gene expression have generated large amounts of data, most of which is freely available in public archives, the use of this valuable resource is limited by computational complications and non-homogenous annotation. To address these issues, we have performed a complete re-annotation of public microarray data from human skeletal muscle biopsies and constructed a muscle expression compendium consisting of nearly 3000 samples. The created muscle compendium is a publicly available resource including all curated annotation. Using this data set, we aimed to elucidate the molecular mechanism of muscle aging and to describe how physical exercise may alleviate negative physiological effects.

**Results:**

We find 957 genes to be significantly associated with aging (*p* < 0.05, FDR = 5 %, *n* = 361). Aging was associated with perturbation of many central metabolic pathways like mitochondrial function including reduced expression of genes in the ATP synthase, NADH dehydrogenase, cytochrome C reductase and oxidase complexes, as well as in glucose and pyruvate processing. Among the genes with the strongest association with aging were H3 histone, family 3B (H3F3B, *p* = 3.4 × 10^−13^), AHNAK nucleoprotein, desmoyokin (AHNAK, *p* = 6.9 × 10^−12^), and histone deacetylase 4 (HDAC4, *p* = 4.0 × 10^−9^). We also discover genes previously not linked to muscle aging and metabolism, such as fasciculation and elongation protein zeta 2 (FEZ2, *p* = 2.8 × 10^−8^). Out of the 957 genes associated with aging, 21 (*p* < 0.001, false discovery rate = 5 %, *n* = 116) were also associated with maximal oxygen consumption (VO_2MAX_). Strikingly, 20 out of those 21 genes are regulated in opposite direction when comparing increasing age with increasing VO_2MAX_.

**Conclusions:**

These results support that mitochondrial dysfunction is a major age-related factor and also highlight the beneficial effects of maintaining a high physical capacity for prevention of age-related sarcopenia.

**Electronic supplementary material:**

The online version of this article (doi:10.1186/s13395-015-0059-1) contains supplementary material, which is available to authorized users.

## Background

Aging profoundly affects skeletal muscle, including loss of muscle mass and strength and increasing the levels of fat and connective tissue [1]. This condition, often termed age-related sarcopenia, leads to a variety of physical conditions that reduce life quality and overall health in aging individuals [2, 3]. As we age, we lose approximately 1 % of leg lean mass per year and approximately 2.5–4 % in leg strength, men to a higher extent than women [4]. This indicates that sarcopenia is not only a matter of loss of muscle mass but rather a concomitant loss of muscle mass and a decline of muscle quality. In order to efficiently delay the onset and severity of sarcopenia, it is crucial to more in detail describe the molecular mechanisms causing this physiological deterioration of muscle function.

In one of the largest previous studies on gene expression in aging muscle [5], muscle biopsies from 81 individuals were investigated. Zahn et al. described a 250-gene signature for muscle aging, compared this to age-associated gene regulation in other tissues and found increased expression of pathways regulating cell growth, complement activation, and ribosomal and extracellular matrix genes and decreased expression of genes for chloride transport and mitochondrial oxidative phosphorylation (OXPHOS). De Magalhaes and colleagues [6] conducted a meta-analysis of microarray experiments on aging in mice, rats, and humans across a variety of tissues. In this cross-species, cross-platform analysis, gene orthologues were meta-analyzed for approximately 400 samples, 42 of which were from human skeletal muscle, comparing old to young individuals. They found 73 genes with altered expression, with increased expression of genes involved in inflammation and immune response, and consistent with Zahn et al. reduced expression of genes associated with energy metabolism, particularly mitochondrial genes (accessible through the GenAge database, http://genomics.senescence.info/genes/). It has also been shown that aging individuals have increasing levels of mitochondrial DNA damage leading to reduced expression of genes in the OXPHOS pathway [7]. Taken together, a general finding is that mitochondrial dysfunction is partly responsible for reduced muscle function with aging [8]. Reduced expression of genes in the OXPHOS pathway, including the regulator peroxisome proliferator-activated receptor gamma coactivator alpha (PGC1α), has also been found to be reduced in skeletal muscle from type 2 diabetic patients [9, 10], a strongly age-related metabolic disorder. Another central pathway previously associated with muscle aging is the mammalian target of rapamycin (mTOR), including the mTOR complex I (mTORC1) which plays a crucial role in the regulation of translation in skeletal muscle [11]. A metabolic link between mTOR and glycolysis has also been described where low glycolytic flux leads to binding of glyceraldehyde-3-phosphate dehydrogenase (GAPDH) to the mTORC1-regulator Rheb thereby inhibiting mTORC1 signaling and suppression of protein synthesis [12].

To understand more the molecular mechanisms of aging in detail, larger sets of samples are required to provide more power to detect regulatory patterns on the gene level. We and others have previously combined data for studies of global transcriptomic patterns across thousands of samples [13–16], but in this study, we address specific phenotype-related questions for skeletal muscle with a collected compendium of 2852 samples. Reuse of public data is however hampered by the use of different experimental platforms and sample annotation, and analysis is not straightforward when combining such data [17].

Based on this muscle expression compendium, we present the largest study to date of gene expression in human skeletal muscle related to aging. We address the concerns of data heterogeneity by an extensive manual re-annotation of all samples and a variety of computational methods described below. In our meta-analysis, we find 957 genes significantly associated with aging. The data provides substantially more detail to gene-specific effects of the transcriptome and shows more widespread regulation of gene expression associated with aging than previously reported. We further study the pleiotropic associations of the 957 genes associated with aging and show for example that 20 out of the 21 aging genes are also associated with physical capacity but regulated in the opposite direction with increased physical capacity as compared to increased age.

The skeletal muscle expression compendium is publicly available at ArrayExpress (http://www.ebi.ac.uk/arrayexpress/) with accession number E-MTAB-1788.

## Methods

### Data collection and annotation

Experiments stored in the ArrayExpress archive [18] were identified by keyword searching aimed at identifying experiments that contained microarrays done on skeletal muscle tissue from living human individuals and with interventions limited to training and glucose/insulin regulation, excluding for example drug treatments. Samples were annotated using the categories and factor values in Table 1.

**Table 1:** Defined terms and value ranges used to annotate the compendium

Parameter	Value	Arrays
Sex	Male	1085
	Female	691
	Mixed	443
	Unknown	85
Age	Age given	993
	Age group	642
	Age range	518
	Unknown age	151
T2D status	Non-T2D	1269
	NGT	321
	IGT	86
	IGT or T2D	89
	T2D	124
	Unknown	415
BMI	BMI given	207
	BMI group	637
	BMI unknown	1460
Physical capacity	PC given	175
	PC group	81
	PC unknown	2048
Family history of diabetes	FH+	24
	FH−	159
	FH unknown	2121
Muscle type	Quad	1970
	Other muscle	242
	Unknown	92
Interventions	Clamp	316
	Longer training	158
	Shorter training/damage	213
	Immobilization 2 days	72
	Immobilization 4–14 days	100
	Immobilization 60 days	170
	Protein intake	88
	Other	196
Immobilized/trained	Trained	108
	Immobilized	175
	Acute trained >24 h	106

2852 samples were annotated. As far as possible, exact values were recorded for numerical parameters. For some studies, individual records were not resolvable, and instead a group average and dispersion measure was given

*T2D* type 2 diabetes

### Preprocessing and quality control

Data normalization was done on the raw .cel files for HG-U133A and HG-U133 + 2 using Robust Probabilistic Averaging (RPA) [19, 20]. Custom array definition files were created using the customCDF R/Bioconductor package (v16), removing probes mapping to known SNPs, and summarizing probes for each gene with an ENSG identifier. Quality control was carried out using the R/Bioconductor package array QualityMetrics [21] removing detected outlier arrays.

### Statistical analysis

Linear regression analysis for the effect of age and physical capacity and removal of study effects was carried out using the limma R/Bioconductor package and the eBayes function. A linear model

$$ x\sim \mathrm{age}+\mathrm{sex}+\mathrm{study} $$

was fitted to the RPA-normalized, gene-summarized data for all genes on each of the two platforms. The “study” parameter was represented by the original ArrayExpress accession number. Model coefficients and *p* values were estimated using the eBayes function in limma. For genes present on both arrays, the minimum *p* value and the maximum of the *β* values for the regression slope of the age parameter were calculated. *p* value correction for false discovery rate (FDR) < 0.05 was done using the Benjamini-Hochberg method using *N* = 31,523 (the total number of *p* values calculated for the two arrays).

To calculate profiles of gene expression as a function of age across the studies, we adjusted the original data for study effects by subtracting the effect quantified in the regression model. For each gene *i* and sample *j,* we calculated

$$ xij={c}_i+{\beta}_{\mathrm{age},i}\times {\mathrm{age}}_j+{\beta}_{\mathrm{sex},i}\times \kern0.5em {\mathrm{sex}}_j+{\varepsilon}_i $$

where *c*_*i*_ is the intercept of the linear regression above, *β*_age_ and *β*_sex_ are the slopes for the age and sex factors, and *ε*_*i*_ is the gene-specific residual from the previous regression.

In the analysis of physical capacity, we used 116 samples from the HG-U133 + 2 array with harmonized annotation for physical capacity, measured as VO_2MAX_ in liters per minute to kilogram (L/(min × kg)). For these, we fitted a linear model

$$ x\sim \mathrm{physical}\kern0.5em \mathrm{capacity}+\kern0.5em \mathrm{Saturday} $$

to the RPA-normalized, gene-summarized expression data. We estimated *p* values and regression coefficients for the model using the limma package with the eBayes function, as above.

We tested the significance of the overlap between subsets of the 957 genes with database lists using the hypergeometric test and a background of *N* = 19,597 genes.

Differential expression between subjects with type 2 diabetes (T2D) and normal glucose tolerance (NGT) individuals for the 957 age-associated genes was estimated by meta-analysis of three datasets with full annotation for these groups: E-GEOD-18732, E-GEOD-19420, and E-GEOD-25462, including 102 T2D and 87 NGT samples. The datasets were individually normalized with RPA and meta-analyzed using the geneMeta R/Bioconductor package (www.bioconductor.org/packages/release/bioc/html/GeneMeta.html). Association to body mass index (BMI) was calculated by retrieving all samples within the seven selected datasets with annotation for BMI from the HG-U133 + 2 arrays and normalized as a single dataset using RPA, followed by a linear regression for BMI adjusted for sex and study, as identified by ArrayExpress accession number and analogously as described for age and physical capacity.

### Comparison with public RNA sequencing data

RNA sequencing expression data on human skeletal muscles from *n* = 157 donors from Genotype-Tissue Expression (GTEx) project (http://www.gtexportal.org/) were used [22]. Across-samples normalization was performed using the TMM normalization method [23]. Association of gene expression for each gene with age was calculated with linear regression using an additive model adjusted for gender. The obtained *p* values were FDR corrected for multiple testing (FDR < 0.05, Benjamini-Hochberg). All calculations were done using R language for statistical computations.

## Results

### Building the skeletal muscle data compendium

From ArrayExpress [18], microarray datasets from human skeletal muscle biopsies were selected and manually curated based on the original publications, including available supplemental data (see “Methods” section). The selected experiments contain data from 2852 microarrays from 20 different array platforms (Figure S1 in Additional file 1). Affymetrix-manufactured arrays dominate, represented by 11 different array types and in total 2475 arrays. Using a controlled vocabulary, sample and experimental parameters selected for re-annotation were defined. We retrieved the original papers along with supplemental material to re-annotate each microarray using our newly defined parameters and their value ranges (Table 1). To define a generic control set, representing a normal, healthy population, a set of 1188 “super controls” were selected. In this group, samples were excluded if the individual had any kind of disease, was obese (BMI > 30), or was subjected to any severe intervention.

To avoid the strong bias introduced by differences in individual probe sequences when combining data from different array platforms [24], we restricted this study to data from each platform independently. We used a subset of the compendium based on the two most common platforms: 568 arrays from the Affymetrix HG-U133A, and 1174 arrays from the HG-U133 + 2 platform. The probe effects were addressed by normalizing each dataset with RPA [19, 20], which models the affinity of each individual probe, assuming it to be a stochastic variable with a normal distribution with probe set-specific mean and variance rather than a constant, as in many other normalization methods including RMA and MAS5. To avoid biases introduced by genetic diversity in the studied individuals, we removed all probes mapping to known human SNPs with a minor allele frequency higher than 5 % in a Western European population. Out of 604,258 probes on the HG-U133 + 2 array, 4840 probes were removed; on the HG-133A array, 2157 out of 247,965 probes were removed. Oligonucleotide probes were summarized to gene level probe sets rather than transcript specific ones, also to minimize biases introduced by probe sequences and their representation on different arrays. After quality control [21], 1236 arrays from the two platforms remained: 758 from HG-U133 + 2 and 485 from HG-U133A. The two resulting data matrices contain data for 19,597 genes tested on the HG-U133 + 2 array and a subset of 11,926 of these on the HG-U133A array. The two resulting cross-study data matrices are also available from ArrayExpress, accession number E-MTAB-1788.

These comprehensive data sets represent comparable human skeletal muscle expression data over a vast array of different experimental conditions. In order to identify constitutively expressed genes, we analyzed the variance of expression only removing the study effect. The genes with most stable expression are presented in Table S1 (see Additional file 1) and are not likely to be influenced by the experimental conditions. The most stable genes found were myoglobin (MB), GAPDH, and alpha 1 actin (ACTA1) and could serve as candidates for “housekeeping” endogenous control genes in quantitative real-time PCR experiments.

### Expression levels of 957 genes are associated with age

**Fig. 1:**
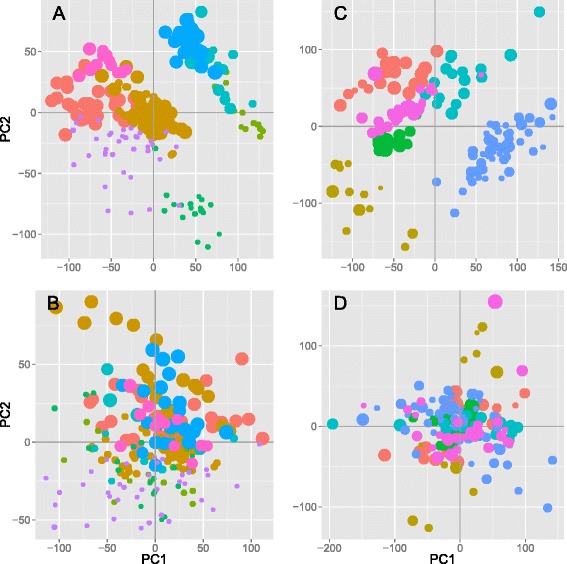
Principal component analysis of the 211 HG-U133A arrays, before (**a**) and after (**b**) removal of study effects and corresponding plots for the 150 HG-U133 + 2 arrays (**c** and **d**). Different studies (identified by database accession numbers) are represented with different colors, and increasing age of the individual from whom the biopsy was taken is indicated by the increasing dot size. The relatively strong study effect seen even after normalization is removed after the adjustment

We selected a subset of 361 arrays from the compendium to study the effect of aging on gene expression, i.e., 211 arrays from the HG-133A array and 150 from the HG-U133 + 2 array that had specific annotation of age and gender, ranging from <1 year up to 83-year-old individuals (Figure S2 in Additional file 1). Using a linear model with sex and study ID as covariates, 957 genes were significantly associated with age (*p* < 0.05, Benjamini-Hochberg correction for multiple testing) (top-50 genes are shown in Table 2). Of these, 484 were upregulated and 473 downregulated with increasing age. We verify the removal of study effects by principal component analysis (PCA) before and after study adjustment. Whereas samples from the same study primarily cluster together in the PCA of the unadjusted dataset, this effect is removed in the adjusted one (Fig. 1). Similarly, we use PCA to verify the absence of gender biases in the dataset after the adjustment for study effects (Figure S3 in Additional file 1). A significant overlap (*N* = 13, *p* = 1.0 × 10^−5^) and complete concordance for the direction of the expression for all 13 genes found in our data set out of the 73 genes detected in the multispecies de Magalhaes study [6] were observed. Twenty-five of the 957 genes are reported in the GenAge database of 288 genes linked to aging, an overlap of the two lists which is significant at *p* = 0.0020 using a hypergeometric test. The GenAge database has also collected and curated a list of genes in loci detected in genome-wide association studies for longevity. They report 886 genes, 353 of which were significantly associated in the original studies. Out of this list, we detect an overlap of 25 out of 957 genes (*p* = 0.024). Seventeen of the 957 aging genes have been previously reported in the top 250 genes by the Zahn study [5] (overlap *p* = 0.065). Using publicly available RNA sequencing data (*n* = 157) from the GTEx project (http://www.gtexportal.org/), 91 genes out of the 957 were found associated with age, a significant overlap at *p* = 2.2 × 10^−16^.

**Table 2:** Top 50 genes significantly associated with age across 361 samples

Gene	Min (*p*)	Max |β|
H3F3B	3.39E-13	0.0098
AHNAK	6.87E-12	0.0086
HOXB2	1.01E-11	−0.0184
CRIM1	5.34E-11	0.0124
NAP1L1P3	9.74E-11	0.0154
ARFGAP2	3.56E-10	0.0069
WDR6	4.47E-10	0.0069
DLEU1	8.61E-10	−0.0117
USP6	1.43E-09	0.0133
TCF25	2.70E-09	0.0064
SCN4B	3.13E-09	−0.0289
ZNF274	3.87E-09	0.0129
HDAC4	4.04E-09	0.0155
SH3BP5	5.04E-09	0.0114
SART3	5.32E-09	0.0050
CASC3	9.73E-09	0.0061
CIRBP	1.14E-08	0.0091
HIST1H2BN	1.49E-08	−0.0065
FUBP1	1.74E-08	0.0058
FAM171A1	1.94E-08	−0.0113
NPPA	1.97E-08	−0.0048
TMEM59L	2.39E-08	−0.0080
UBE2O	2.79E-08	−0.0060
FEZ2	2.83E-08	0.0126
ALS2CL	3.15E-08	−0.0063
C1S	3.22E-08	0.0137
RXRG	3.23E-08	−0.0095
NT5C2	3.64E-08	0.0115
TRMT112	4.42E-08	0.0116
PRNP	4.68E-08	0.0094
NOL9	6.31E-08	0.0049
RBM10	6.66E-08	0.0056
ANP32B	6.71E-08	0.0092
TOMM40L	6.74E-08	−0.0144
HTR5A	7.21E-08	−0.0051
SEC24C	7.78E-08	0.0060
BUB3	7.98E-08	0.0036
DAAM2	8.92E-08	−0.0091
HSPA1A	8.93E-08	0.0091
SEZ6L2	9.12E-08	−0.0046
MRPL4	9.38E-08	−0.0080
CMC2	1.04E-07	−0.0091
NR1D1	1.04E-07	−0.0054
ENDOG	1.11E-07	−0.0126
MRPL48	1.25E-07	−0.0066
FRAT2	1.31E-07	−0.0064
SYNRG	1.31E-07	0.0040
PPIC	1.38E-07	−0.0076
POMT1	1.47E-07	0.0061
DECR2	1.65E-07	0.0043

The minimum *p* and maximum *β* values were calculated for genes present in both array datasets, adjusted for sex and study effect. A positive *β* value implicates increasing gene expression with age. For the full list of 957 genes, see Table S2 in Additional file 2

The genes with the strongest association to aging in the current study are H3F3B (*p* = 3.4 × 10^−13^) and AHNAK (*p* = 6.9 × 10^−12^), both showing increased expression with increased age. AHNAK is a large protein localized to the sarcomere of skeletal muscle and increased expression of AHNAK with aging is in line with the study by de Magalhaes et al. [6]. Increased expression of AHNAK has previously also been associated with a low VO_2MAX_ and poor muscle fitness [25]. The two genes most strongly showing reduced expression with increasing age are homeobox B2 (HOXB2, *p* = 1.0 × 10^−11^) and deleted in lymphocytic leukemia 1 (DLEU1, *p* = 8.6 × 10^−10^). The full list of 957 genes is available in Table S2 (see Additional file 2).

### Aging significantly alters genes involved in inflammation and mitochondrial metabolism

We analyzed the lists of 484 genes upregulated and 473 downregulated with aging for enrichment of specific pathways and processes using gene set enrichment analysis (GSEA) (Table 3) [9]. We find enrichment of genes with increased expression in six pathways, connected to the complement system, indicating an inflammatory response with aging (*p* < 0.05, Bonferroni adjusted). This is in line with Zahn et al. that also reported increased expression of genes in the complement system with aging [5]. Thirteen pathways were enriched in genes with decreased expression associated with increasing age (*p* < 0.05, Bonferroni adjusted). Eight of these represent mitochondrial components, supporting a perturbation of mitochondrial function with aging. Four groups connected to metabolism were also found, indicating reduced expression of genes in the electron transport chain (ETC)/OXPHOS pathway, in the citric acid/tricarboxylic acid cycle (TCA), and in pyruvate metabolism. We observe significant downregulation with increasing age of all four major complexes of the ETC located in the inner mitochondrial membrane (Figure S4 in Additional file 1). Subunits of NADH dehydrogenase (NDUFAF5, *p* = 1.0 × 10^−4^; NDUFS3, *p* = 8.8 × 10^−5^), cytochrome c reductase (UQCR10, *p* = 2.6 × 10^−4^; UQCR11, *p* = 7.7 × 10^−4^) and oxidase (COX10, *p* = 4.3 × 10^−5^; COX4I1, *p* = 1.7 × 10^−4^; COX7B, *p* = 3.1 × 10^−4^; COX7C, *p* = 6.1 × 10^−4^; COX5B, *p* = 1.1 × 10^−3^), and ATP synthase (ATP5G3, *p* = 1.1 × 10^−6^; ATPAF1, *p* = 3.6 × 10^−5^; ATP5G1, *p* = 8.0 × 10^−5^; ATP5C1, *p* = 2.0 × 10^−4^; COX10, *p* = 4.3 × 10^−5^) are all downregulated with increasing age. We also find that the expression of C-x(9)-C motif containing 2 (CMC2) is decreased with aging (*p* = 1.0 × 10^−7^). CMC2 is required for respiratory growth, and mutants with CMC2 deletion are unable to assemble the cytochrome c oxidase complex [26]. In the pyruvate dehydrogenase complex (PDC), proteins A1, B, and X (*p* = 4.1 × 10^−6^; 4.2 × 10^−5^; 1.3 × 10^−3^) show reduced expression with aging. In connection to carbohydrate metabolism, the expression of 6-phosphofructo-2-kinase/fructose-2,6-biphosphatase 1 (PFKFB1) is increased with aging (*p* = 9.4 × 10^−5^). Furthermore, glucose uptake through GLUT4 in skeletal muscle is known to be dependent on TBC1 Domain Family Member 1 (TBC1D1) [27, 28], the expression of which is decreased with aging (*p* = 3.6 × 10^−4^).

**Table 3:** Gene set enrichment analysis for the 484 genes upregulated with age and the 473 downregulated

Upregulated sets	ES	NES	FDR *q* value
KEGG_COMPLEMENT_AND_COAGULATION_CASCADES	0.80	2.32	0.0047
REACTOME_INITIAL_TRIGGERING_OF_COMPLEMENT	0.89	2.18	0.0133
REACTOME_COMPLEMENT_CASCADE	0.89	2.15	0.0138
BIOCARTA_COMP_PATHWAY	0.86	2.21	0.0148
KEGG_SYSTEMIC_LUPUS_ERYTHEMATOSUS	0.75	2.07	0.0288
BIOCARTA_CLASSIC_PATHWAY	0.90	2.04	0.0361
Downregulated sets			
REACTOME_TCA_CYCLE_AND_RESPIRATORY_ELECTRON_TRANSPORT	−0.76	−2.90	0.0000
MITOCHONDRION	−0.59	−2.70	0.0000
REACTOME_PYRUVATE_METABOLISM_AND_CITRIC_ACID_TCA_CYCLE	−0.81	−2.48	0.0008
MITOCHONDRIAL_ENVELOPE	−0.74	−2.39	0.0010
MITOCHONDRIAL_MEMBRANE	−0.74	−2.37	0.0011
MITOCHONDRIAL_PART	−0.74	−2.39	0.0012
KEGG_PARKINSONS_DISEASE	−0.73	−2.40	0.0013
ORGANELLE_INNER_MEMBRANE	−0.74	−2.30	0.0014
ORGANELLE_ENVELOPE	−0.68	−2.33	0.0015
MITOCHONDRIAL_INNER_MEMBRANE	−0.74	−2.30	0.0015
ENVELOPE	−0.68	−2.26	0.0019
KEGG_OXIDATIVE_PHOSPHORYLATION	−0.64	−2.23	0.0024
REACTOME_PYRUVATE_METABOLISM	−0.81	−2.22	0.0025
COFACTOR_METABOLIC_PROCESS	−0.69	−2.19	0.0037
KEGG_ALZHEIMERS_DISEASE	−0.60	−2.07	0.0117
MITOCHONDRIAL_MEMBRANE_PART	−0.74	−2.08	0.0123
REACTOME_RESPIRATORY_ELECTRON_TRANSPORT_ATP_SYNTHESIS_BY_CHEMIOSMOTIC_COUPLING_AND_HEAT_PRODUCTION_BY_UNCOUPLING_PROTEINS_	−0.70	−2.02	0.0161
REACTOME_GLUCOSE_METABOLISM	−0.70	−2.03	0.0162
REACTOME_GLUCONEOGENESIS	−0.77	−2.01	0.0187
ION_TRANSPORT	−0.82	−1.99	0.0199
COENZYME_METABOLIC_PROCESS	−0.82	−2.00	0.0200
REACTOME_REGULATION_OF_PYRUVATE_DEHYDROGENASE_PDH_COMPLEX	−0.81	−1.96	0.0246
HYDROGEN_ION_TRANSMEMBRANE_TRANSPORTER_ACTIVITY	−0.78	−1.94	0.0283
INORGANIC_CATION_TRANSMEMBRANE_TRANSPORTER_ACTIVITY	−0.78	−1.91	0.0340
ION_TRANSMEMBRANE_TRANSPORTER_ACTIVITY	−0.50	−1.90	0.0378
MONOVALENT_INORGANIC_CATION_TRANSMEMBRANE_TRANSPORTER_ACTIVITY	−0.78	−1.89	0.0380
KEGG_CITRATE_CYCLE_TCA_CYCLE	−0.77	−1.89	0.0385

### High physical capacity counteracts age-related changes in muscle expression

Decline in muscle mass and performance with aging can be prevented by exercise. Therefore, we explored whether expressions of any of the genes associated with aging also were influenced by physical capacity assessed as VO_2MAX_.

**Fig. 2:**
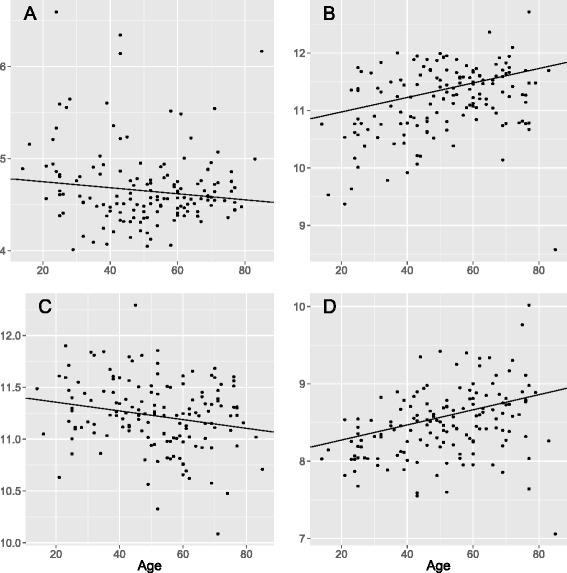
Expression levels of SOCS2 (**a**), FEZ2 (**b**), MPC1 (**c**), and NT5C2 (**d**) in relation to age. Batch effect-adjusted expression levels are shown

In a subset of 116 samples with information on VO_2MAX_, we find 39 genes associated with physical capacity (FDR < 0.05, Benjamini-Hochberg, Table 4), but given the relatively low number of samples included in the VO_2MAX_ analysis, we restricted our analysis to the 957 genes associated with age. Of them, 21 were also associated with VO_2MAX_ (Table 5). It is striking, but not unexpected, that aging and increased physical capacity affects gene expression in opposite directions for 20 of the 21 genes. Two of these, the suppressor of cytokine signaling 2 (SOCS2) and the fasciculation and elongation protein zeta 2 (FEZ2) are also associated with BMI (FEZ2: *p*_BMI_ = 5.9 × 10^−4^, SOCS2: *p*_BMI_ = 7.3 × 10^−4^); increasing BMI affects gene expression in the same direction as increasing age (FEZ2: *p*_age_ = 2.8 × 10^−8^, SOCS2: *p*_age_ = 5.5 × 10^−7^) and in the opposite direction with increased physical capacity (FEZ2: *p*_VO2max_ = 3.5 × 10^−5^, SOCS2: *p*_VO2max_ = 6.3 × 10^−8^) (Fig. 2).

**Table 4:** Thirty-nine genes genome-wide significantly associated with physical capacity across 116 samples

Gene	Min (*p*)	Max |β|
SOCS2	6.28E-08	0.014
SLC16A10	8.53E-07	−0.024
HOMER1	1.02E-06	−0.018
ZMYND17	1.84E-06	−0.021
BCKDHB	3.06E-06	0.010
INADL	4.99E-06	−0.011
MAST2	5.28E-06	−0.011
BDH1	5.76E-06	0.015
ZNF133	6.37E-06	−0.008
METTL7A	1.22E-05	−0.022
ZNF57	1.30E-05	0.011
TMEM56	1.61E-05	−0.021
RALGAPA1	1.66E-05	−0.010
CALU	1.89E-05	0.009
SYNPO2L	2.11E-05	−0.014
RP11-304 L19.5.1	2.33E-05	−0.018
AQP1	2.79E-05	0.014
SCPEP1	2.96E-05	−0.014
KIAA1109	3.02E-05	0.004
HYI	3.46E-05	−0.008
FEZ2	3.46E-05	−0.020
EIF4E2	3.79E-05	−0.016
LPL	3.85E-05	0.028
ZNRF1	3.89E-05	−0.010
YPEL2	3.98E-05	−0.013
DMRT2	4.37E-05	0.019
UGGT1	4.61E-05	−0.007
MPP7	4.79E-05	0.012
SUN1	5.37E-05	−0.008
BRD8	5.77E-05	−0.008
PRKAG3	6.04E-05	−0.025
SLC38A1	6.10E-05	0.047
ITGA6	6.32E-05	0.018
CMBL	6.74E-05	−0.011
NANOS1	8.65E-05	−0.035
SCGB1D2	8.80E-05	0.027
MESP1	8.97E-05	−0.008
HSPA2	9.31E-05	−0.022
HEMK1	9.56E-05	−0.008

A positive *β* value implicates increasing gene expression with increased physical capacity, adjusted for study effect

**Table 5:** Of the 957 aging genes, 21 were significantly associated with physical capacity across 116 samples

Gene	*p* (age)	*p* (PC)	*β* (age)	*β* (PC)
SOCS2	5.51E-07	6.28E-08	−0.0034	0.0143
SLC16A10	0.000785	8.53E-07	0.00354	−0.02369
METTL7A	0.001265	1.22E-05	0.011168	−0.02183
CALU	0.00086	1.89E-05	−0.00311	0.009051
FEZ2	2.83E-08	3.46E-05	0.012589	−0.01958
DMRT2	0.000223	4.37E-05	−0.01035	0.018518
ITGA6	0.000564	6.32E-05	−0.00607	0.017743
MESP1	0.000246	8.97E-05	−0.00638	−0.00767
IP6K2	2.32E-06	0.000112	0.005743	−0.00807
ANKRD27	0.000118	0.000121	0.006005	−0.02111
MPC1	0.000705	0.000148	−0.00478	0.010666
PAF1	1.07E-06	0.000258	0.006852	−0.01292
SLIT2	0.000698	0.000297	0.009127	−0.01342
DDX24	2.01E-05	0.000318	0.004313	−0.00869
FAM53C	0.001351	0.000342	0.003416	−0.00845
CPSF7	0.000491	0.000405	0.003669	−0.00789
DGKD	0.000456	0.000838	0.00429	−0.01153
PHF20	0.001338	0.000871	0.00284	−0.00665
ABRA	0.001011	0.000952	0.014313	−0.01971
NT5C2	3.64E-08	0.000954	0.011521	−0.01262
DNAJB2	9.22E-07	0.000956	0.007196	−0.01217

A positive *β* value implicates increasing gene expression with increased physical capacity, adjusted for study effect

## Effect of body mass index and type 2 diabetes on the expression of age-associated genes

We find that three of the age-associated genes also were associated with T2D, CD163 (*p*_age_ = 2.2 × 10^−4^, *p*_T2D_ = 2.0 × 10^−4^), ZNF415 (*p*_age_ = 8.9 × 10^−5^, *p*_T2D_ = 8.5 × 10^−5^), and GADD45A (*p*_age_ = 5.4 × 10^−4^, *p*_T2D_ = 1.1 × 10^−4^) (Table 6). Of these, GADD45A and CD163 have previously been shown to be associated with T2D in GWAS [29, 30]. Interestingly, GADD45A has also been shown to reduce energy production and to stimulate pro-atrophy mechanisms in skeletal muscle [31]. ZNF415 is known to inhibit AP1 and p53 transcriptional activity [32], whereas increased concentration of serum sCD163 is a risk factor for developing T2D [33]. Two of the 957 age-associated genes were also associated with BMI, i.e., EIF4EBP1 (*p*_BMI_ = 1.8 × 10^−6^) and AKR1C3 (*p*_BMI_ = 4.7 × 10^−5^).

**Table 6:** Of the 957 aging genes, three were associated with type 2 diabetes

Gene	*p* (age)	*p* (T2D)	*β* (age)	*Z* (T2D)
CD163	0.00022	0.000195	0.009341	3.725788
ZNF415	8.93E-05	8.49E-05	−0.01078	−3.9301
GADD45A	0.000541	0.000112	0.011251	3.862089

Analysis of 102 samples with type 2 diabetes (T2D) versus 87 normoglycemic individuals. A positive *Z* value implicates increasing gene expression with T2D, adjusted for study effect

## Discussion

Because of differences in annotation standards, analysis of public gene expression data is often hampered by an inability to combine large sets of arrays for new studies. A major benefit of the data set presented here is that it has been manually annotated using a harmonized vocabulary, enabling a more comprehensive and detailed analysis to be performed. We show that through a strong manual curation effort, we could increase the combinability and utility of public data, deriving the until now largest study on aging in human skeletal muscle, and from the same compendium address additional questions regarding physical capacity, BMI, and T2D. This skeletal muscle compendium is publicly available to allow further studies on gene expression in skeletal muscle.

**Fig. 3:**
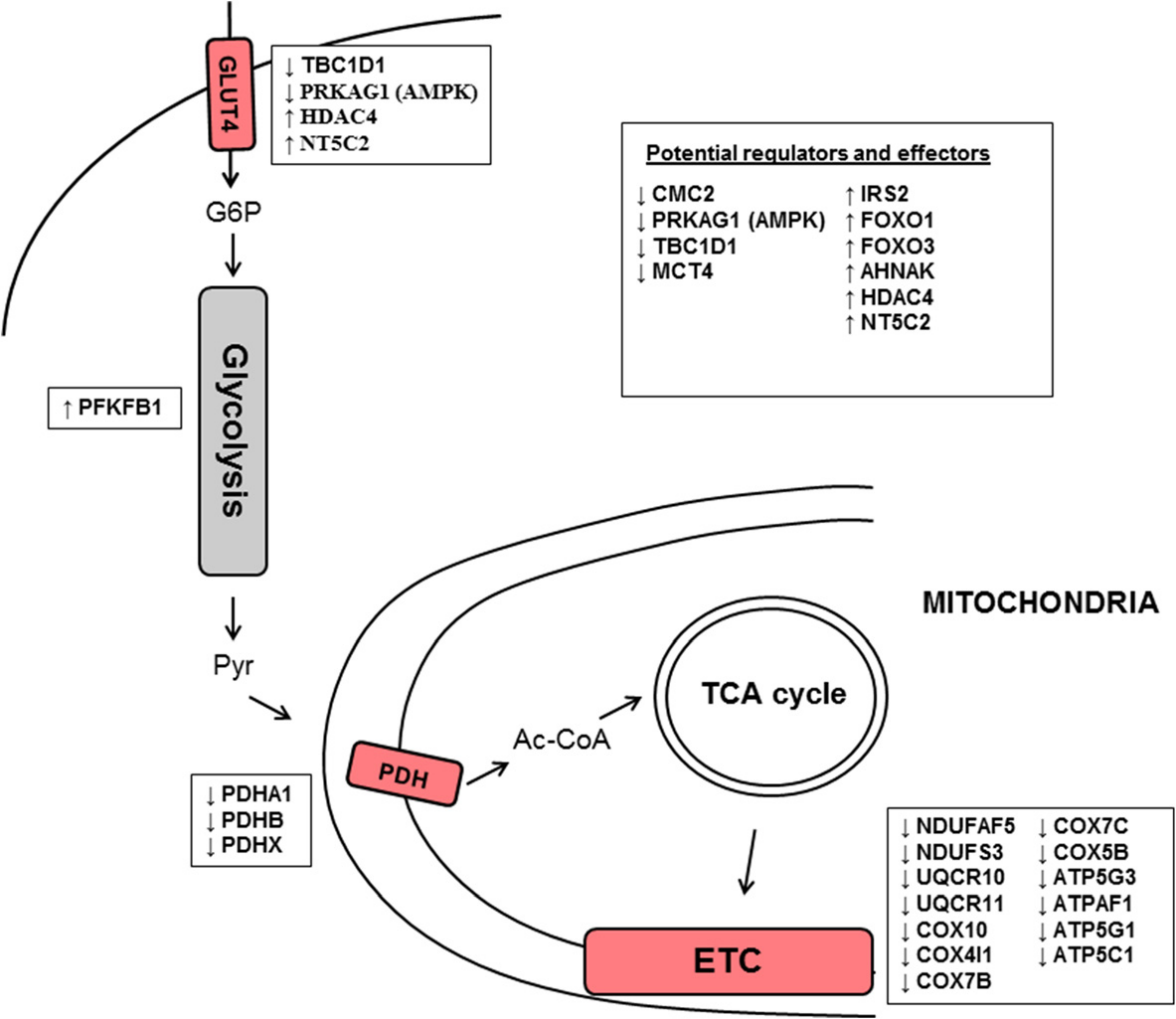
Schematic illustration of major metabolic effects of aging in human skeletal muscle. A subjectively selected set of effector and regulatory genes from the 957 age-associated genes are shown with their direction of regulation shown with respect to increasing age

In the current analysis, we present a detailed description of age-related differences in gene expression in human skeletal muscle and identify 957 genes significantly associated with age. In line with Zahn et al., we find that genes in the complement system show increased expression and mitochondrial genes show decreased expression with aging [5]. Expression of genes in all the major complexes in the ETC, as well as several genes in the PDH complex decreased with aging (Fig. 3). These results together with those of others [34, 35] support the view that elderly subjects have a nearly 50 % lower oxidative capacity per volume of muscle than younger subjects [36]. At the cellular level, this decrease has been ascribed to a reduction in mitochondrial content and lower oxidative capacity of the mitochondria [36], i.e., this decrease of mitochondrial constituents could either reflect defective mitochondria or decreased number of mitochondria or both. Several potential regulators of mitochondrial mass and function were identified among the 957 age-associated genes in the current study. For example, ENDOG is a protein regulated by PGC1A, shown to interact with the mitochondrial genome to regulate mitochondrial mass [37]. TOMM40 is a crucial subunit of the translocase responsible for import of nuclear-encoded mitochondrial precursor proteins [38], which has previously been associated with aging and with exercise-induced mitochondrial biogenesis [39]. MRPL4 and MRPL48 are components of the mitochondrial ribosome, responsible for the production of essential oxidative phosphorylation proteins and CMC2 is required for mitochondrial cytochrome c oxidase assembly [26]. HCCS and TFRC are other proteins associated with aging that are required for proper functioning of the ETC [40, 41]. Among other potential regulators associated with aging are two forkhead transcription factors, i.e., FOXO1 and FOXO3. Both of these have also previously been implicated for roles in aging, longevity, and muscle atrophy [42, 43]. In summary, deterioration in skeletal muscle mitochondrial function is already well recognized as a major factor contributing to age-related muscle degeneration [34, 35], and our findings support this claim on a broad molecular level, identifying a large number of potential regulators.

We find that genes that have a function in glucose uptake and energy sensing are strongly affected by aging. For example, we see reduced expression of the γ1 regulatory unit of AMPK with increased age. AMPK is a major energy sensor in skeletal muscle, controlling crucial steps of both glucose and lipid metabolism through the ability to sense AMP levels [44]. Reduced AMPK expression is known to result in lower ability of the muscle to utilize glucose through the GLUT4 transporter and to reduce the effectiveness of exercise as a stimulant of glucose uptake and ATP generation through glycolysis, with negative effects on glycemic control and regeneration of muscle mass. Induction of NT5C2 expression with increased age is a possible explanation to the age-associated reduction in AMPK activity, which in turn could be an important contributing factor to reduced mitochondrial function associated with aging [45]. Silencing of NT5C2 expression in cultured human myotubes increased the AMP/ATP ratio and AMPK activity and promoted palmitate oxidation and glucose transport [46], and endogenous expression of NT5C2 is known to inhibit basal lipid oxidation and glucose transport in skeletal muscle. AMPK, in turn, appears to regulate GLUT4 expression via the HDAC4/5-MEF2 axis [47], and in this study, we detected an increased expression of HDAC4 with increased age. The importance of the regulation of GLUT4 levels by AMPK in skeletal muscle is supported by this study showing regulated levels of NT5C2 with age, which can be reversed by physical capacity. Together, the interactions between age-associated changes in gene expression in these key pathways may explain the reduced ability to both generate energy for muscle contraction during exercise and to utilize circulating glucose in the aging muscle.

### Muscle aging and physical capacity

Strikingly, we find that genes that are associated with both aging and physical capacity are largely counteracting. The presented data thereby support efforts to maintain high physical fitness in an aging population to counteract negative effects on mitochondrial function [48]. In particular, we hypothesize that SOCS2 and FEZ2, which show significant associations with age, BMI, and physical capacity and acting in the same direction for BMI and age but in the opposite direction for increasing physical capacity, have key regulatory functions in processes that link these three factors. SOCS2 interacts strongly with the activated IGF1R and may play a regulatory role in IGF1 receptor signaling [49]. Age-associated difference in the mRNA level of SOCS2 has previously been demonstrated in muscle from rat, where it was suggested to reflect resistance to the effect of growth hormone [50]. Also, an acute bout of resistance exercise is capable of upregulating SOCS2 in human skeletal muscle [51]. FEZ2 is to our knowledge a novel age-associated gene, the expression of which was altered in the opposite direction with physical capacity.

## Conclusions

We show that through a strong manual curation effort, we could increase the combinability and utility of public data, deriving the until now largest study on aging in human skeletal muscle. This skeletal muscle compendium is publicly available, with applications for further studies on transcriptional regulation in skeletal muscle for a number of physiological and biological questions. Overall, our results paint a convoluted picture with many age-related pathways affecting a wide range of fundamental cellular processes. These results support that mitochondrial dysfunction is a major age-related factor and also highlight the beneficial effects of maintaining a high physical capacity for prevention of age-related sarcopenia.

## Additional files

Additional file 1: Supplementary material FigureS S1-4 and Table S1 (DOCX 385 kb)

Additional file 2: Table S2 957 genes significantly associated with age across 340 samples (XLSX 257 kb)

## Abbreviations

ACTA1: alpha 1 actin
AHNAK: AHNAK nucleoprotein, desmoyokin
CMC2: C-x(9)-C motif containing 2
DLEU1: deleted in lymphocytic leukemia 1
ETC.: electron transport chain
FDR: false discovery rate
FEZ2: fasciculation and elongation protein zeta 2
GAPDH: glyceraldehyde-3-phosphate dehydrogenase
GSEA: gene set enrichment analysis
H3F3B: H3 histone, family 3B
HOXB2: homeobox B2
MB: myoglobin
mTOR: mammalian target of rapamycin
mTORC1: mTOR complex I
NGT: normal glucose tolerance
OXPHOS: mitochondrial oxidative phosphorylation
PCA: principal component analysis
PDC: pyruvate dehydrogenase complex
PFKFB1: 6-phosphofructo-2-kinase/fructose-2,6-biphosphatase 1
PGC1α: peroxisome proliferator-activated receptor gamma coactivator alpha
RPA: robust probabilistic averaging
SOCS2: suppressor of cytokine signaling 2
T2D: type 2 diabetes
TBC1D1: TBC1 domain family member 1
TCA: tricarboxylic acid cycle
VO_2MAX_: maximal oxygen consumption
