# Supplementary material for: A novel atlas of gene expression in human skeletal muscle reveals molecular changes associated with aging
Source: Skelet Muscle. 2015 Oct 9;5:35. doi: 10.1186/s13395-015-0059-1 (PMC4600214; doi:10.1186/s13395-015-0059-1)
Supplement: Supplementary file 1 — FigureS S1-4 and Table S1 (DOCX 385 kb) [file 13395_2015_59_MOESM1_ESM.docx]

A novel atlas of expression in human skeletal muscle reveals molecular changes associated with aging

Supplemental material

ArrayExpress

37.390 experiments

>1,000,000 arrays

Manual selection and annotation

Muscle Compendium

77 experiments

2,852 arrays

HG-U133A/+2 arrays

39 experiments

1,655 arrays

QC’d arrays

31 experiments

1,236 arrays

Quality control

**Supplemental figure 1.** A compendium of 2,852 public gene expression arrays for skeletal muscle was collected, quality controlled, pre-processed and manually re-annotated. The use of a harmonized vocabulary and the large number of samples facilities the use of the compendium for a wide range of topics concerning gene expression in skeletal muscle. From this compendium, we selected the sample subsets for age, sex and physical capacity analysis. Above, we show the selection pipeline for the sample subset used in the age analysis.


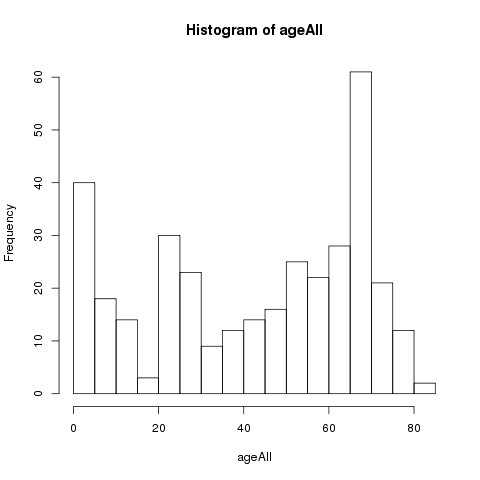


**Supplemental figure 2.** The 359 samples selected for the age study ranged from < 1 to 83 years of age, with slight focus on the very young and the age group 60-70 years.

**
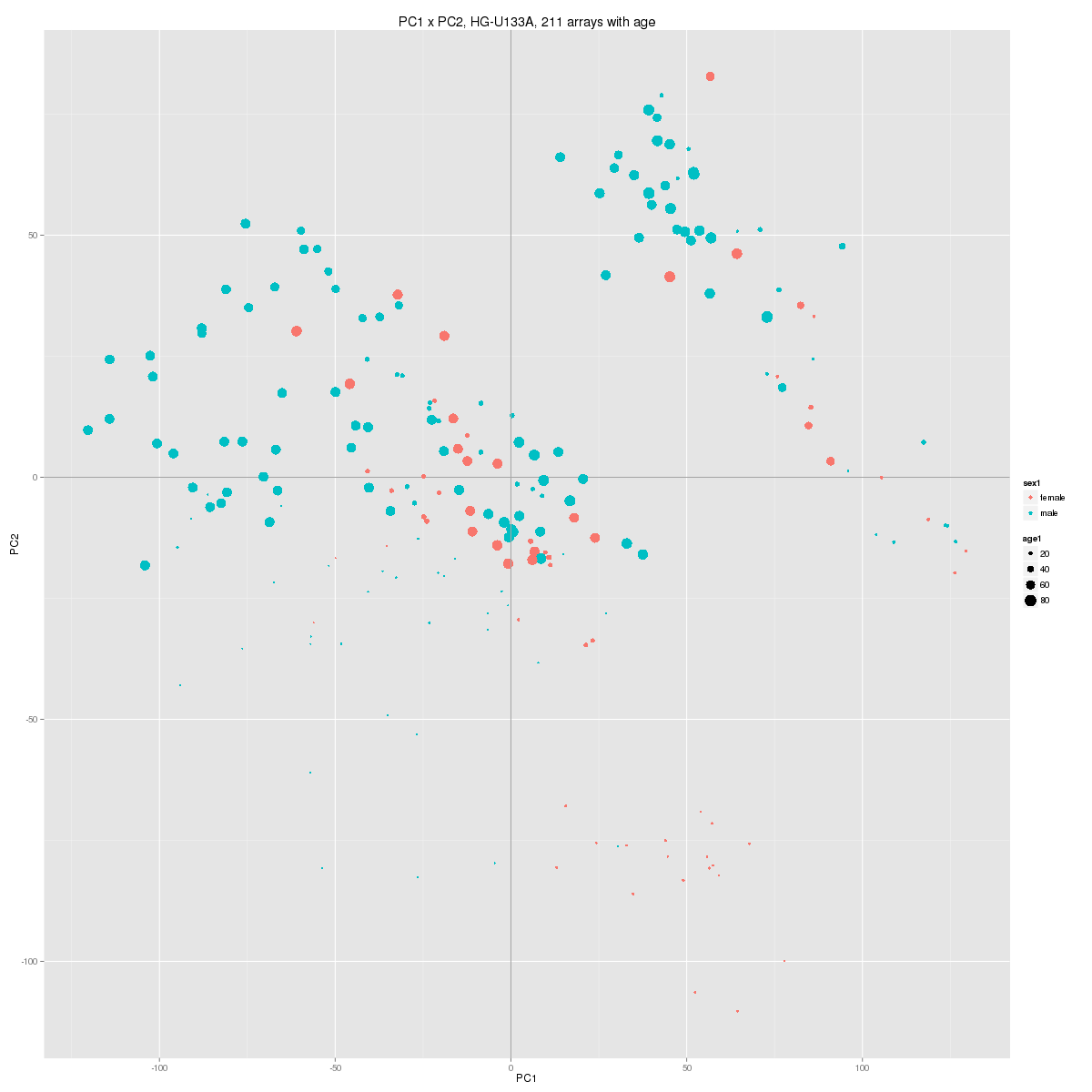
Supplemental figure 3.** Principal component analysis of the 211 HG-U133A arrays, before (A) and after (B) removal of study effects, and corresponding plots for the 150 HG-U133+2 arrays (C and D). Samples from male individuals are coloured blue, and female red. Increasing age of the individual from whom the biopsy was taken is indicated by the increasing dot size. We do not observe any gender bias before or after adjustment for batch effects.

**D**

**C**

**B**

**A**


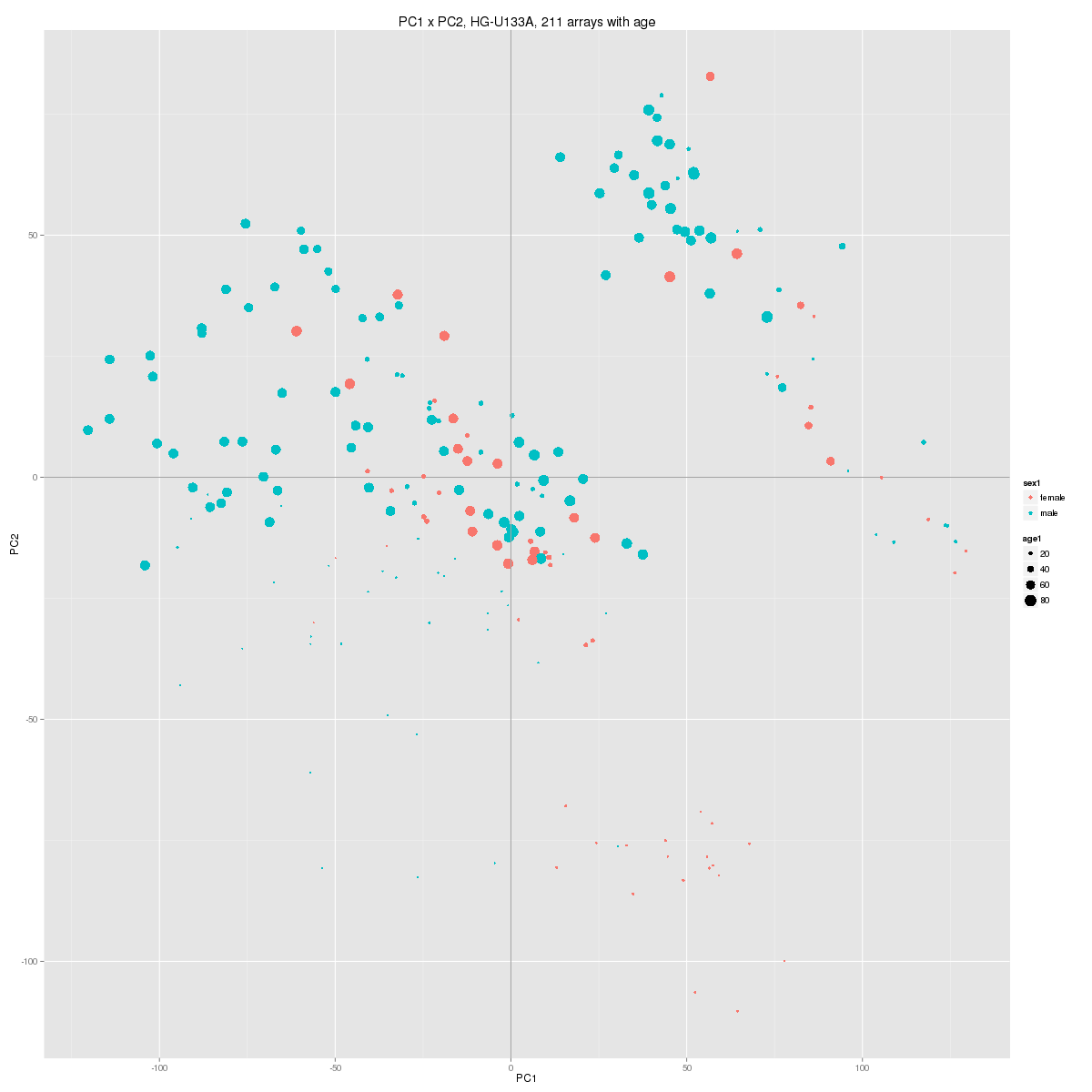

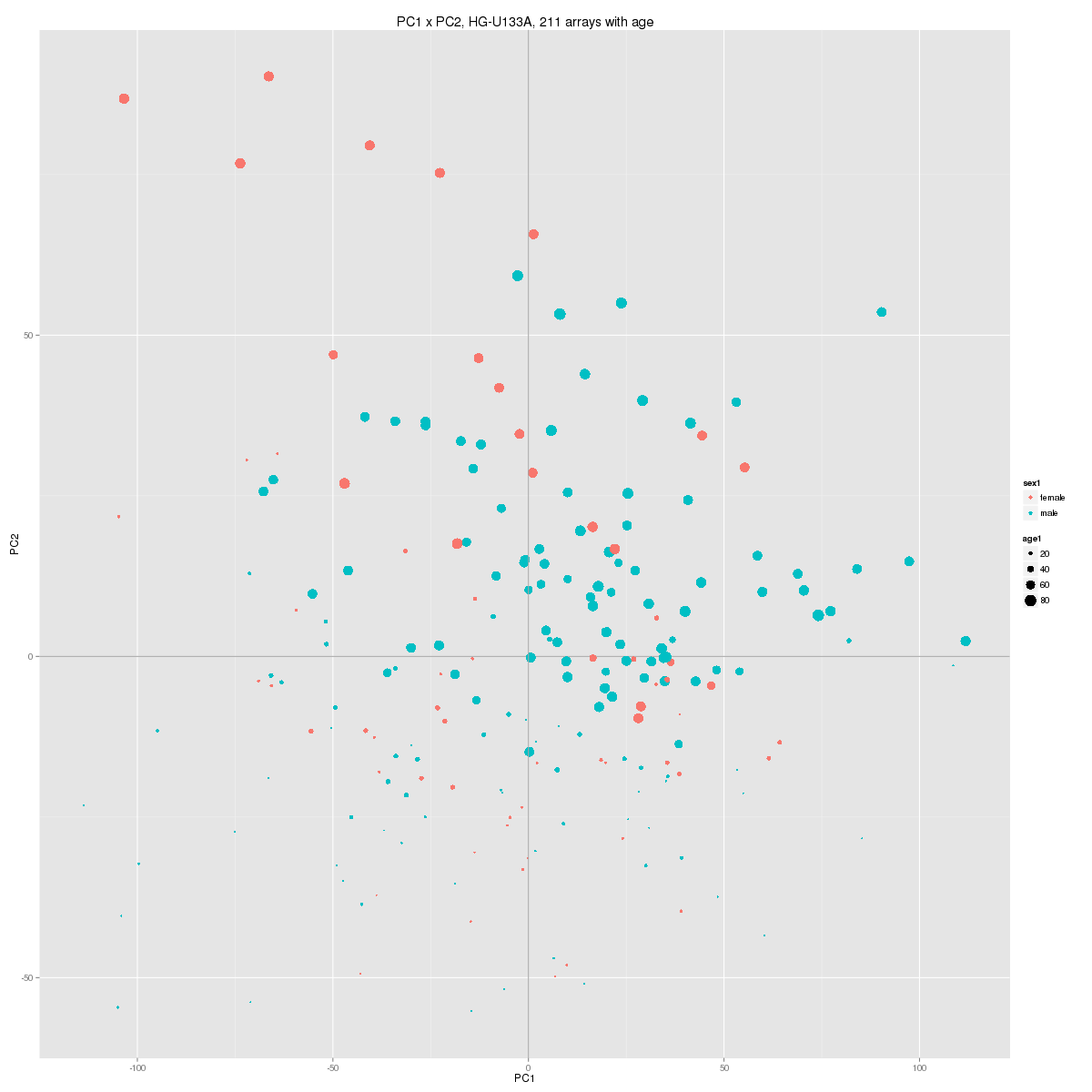

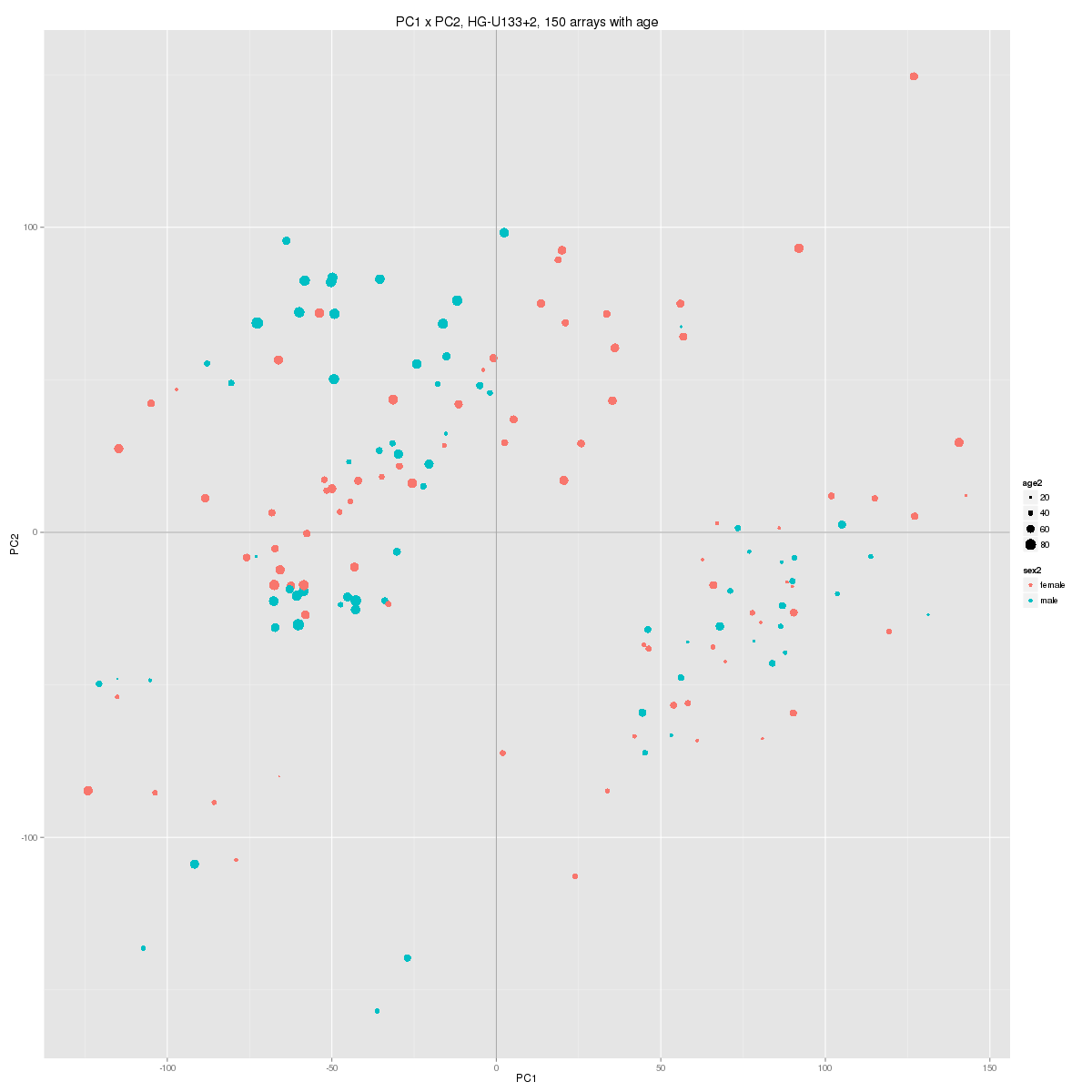

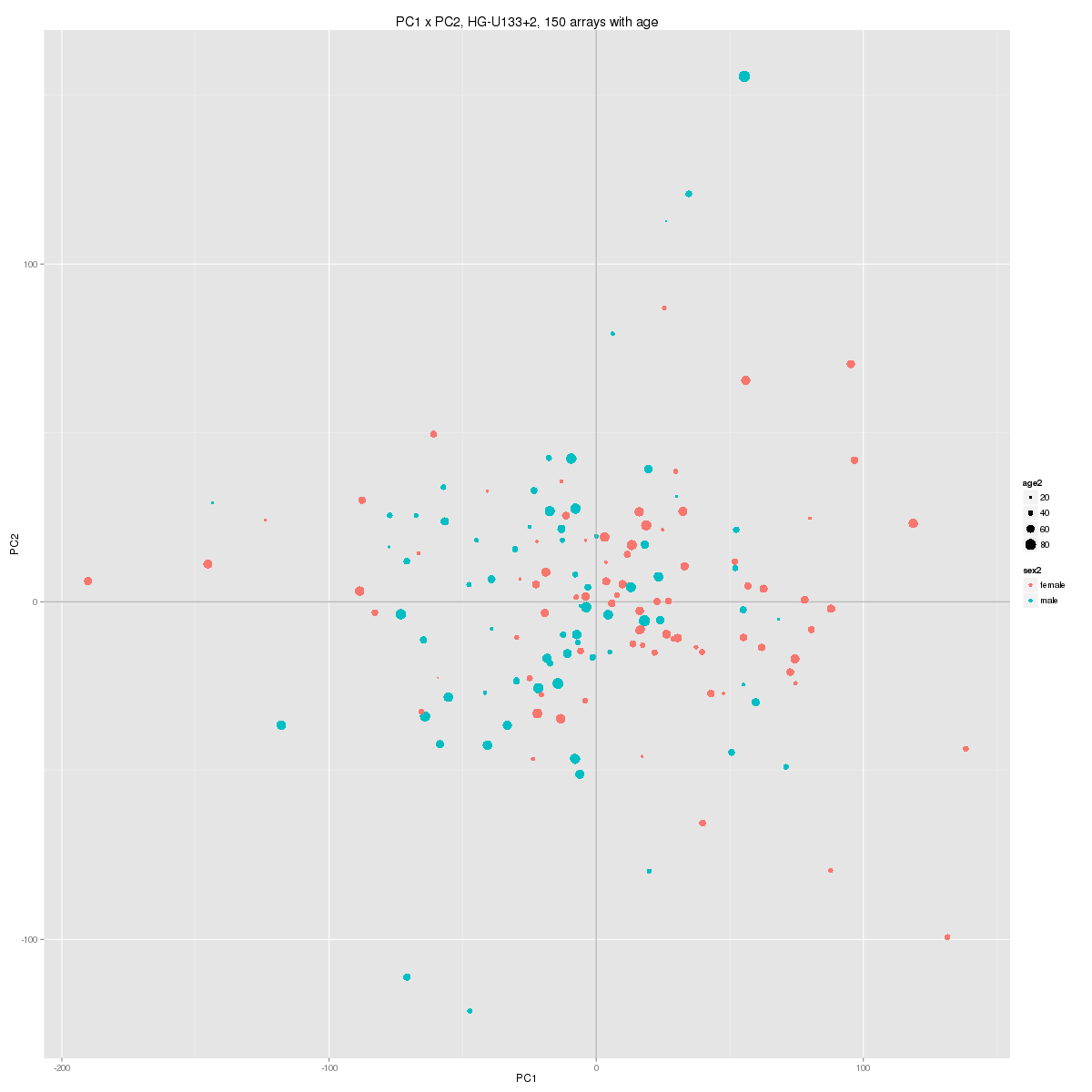


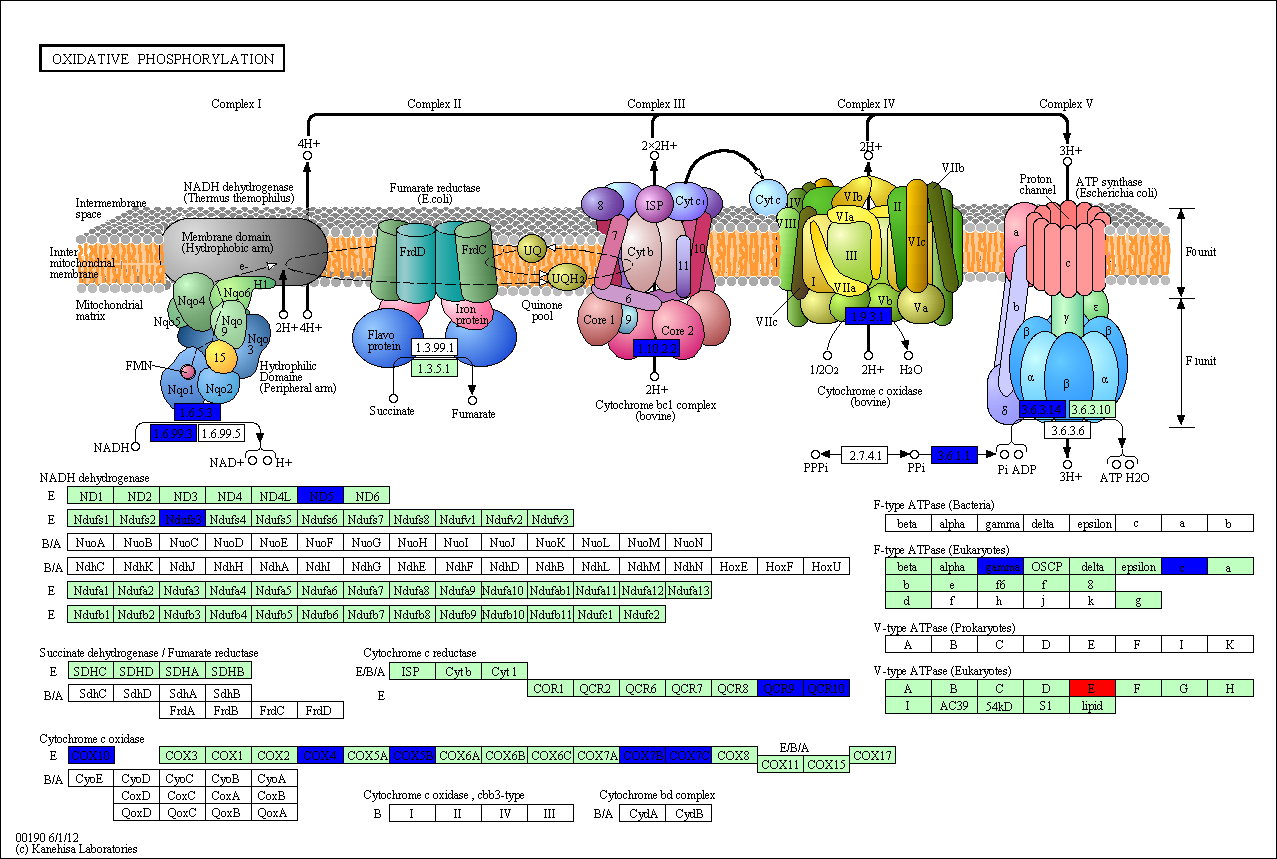


**Supplemental figure 4.** All human core components of the oxidative phosphorylation pathway are downregulated (blue) in aging skeletal muscle, reducing mitochondrial function and the cell’s ability to produce ATP.

| Supplemental table 1. The 10 most stably expressed genes across 872 HG-U133+2 arrays run on skeletal muscle samples, after correction for study effect. | |
| --- | --- |
| Gene | CV |
| MB | 0.000445851 |
| GAPDH | 0.000510746 |
| ACTA1 | 0.000657875 |
| CKM | 0.000747423 |
| MYBPC1 | 0.000791782 |
| MT-ND4 | 0.001018796 |
| TTN | 0.001223888 |
| NDUFB4 | 0.001337269 |
| ERICH1 | 0.001396224 |
| FKBP15 | 0.001404073 |
| CV is the coefficient of variation, calculated as the variance over all arrays divided by the mean value | |
